# The Impact of the Hippocratic Oath in 2018: The Conflict of the Ideal of the Physician, the Knowledgeable Humanitarian, Versus the Corporate Medical Allegiance to Financial Models Contributes to Burnout

**DOI:** 10.7759/cureus.3076

**Published:** 2018-07-30

**Authors:** Sharon A Clark

**Affiliations:** 1 Plastic Surgery/chief, Mills-Peninsula Medical Center, San Mateo, USA

**Keywords:** hippocratic oath, ethics, economics, mba oath, burnout, pledge, profession of medicine, trade, safety, declaration of geneva

## Abstract

The tradition in medical school includes taking the Hippocratic Oath usually at graduation. The purpose of this review is to examine what that oath has been, what forms it currently has, and the implications for physicians in today’s healthcare environment. The changes in health economics affect physicians as they try to follow the oath’s allegiance to the individual patient’s needs. At times, this goal conflicts with the perspective of the financial world’s controls of insurance companies and medical groups and institutions. This difference of the physicians’ ethical perspectives from the business leaders regarding the philosophy of the value of the individual’s health and life may be related to some aspect of physician burnout.

## Introduction and background

Many populations in the world know of the Hippocratic Oath for physicians as they begin the journey to care for patients. In this current era of medicine the frequency of students' taking the oath has increased to nearly every one compared to the early twentieth century; however, few medical students and physicians actually know that the translations of the ancient words have become less complete, as well as quite varied from the classical translations. With more and more medical students taking an oath, the content actually has been simultaneously thinned. Certainly, the part addressed to faith in the Greek deities, in whom the ancient physicians believed, does not exactly apply for different locations and religions. It does honor the history of medicine and the bond with principles of the selfless tradition of healing. Now the act of saying the oath with peers has been viewed as a process of getting the diploma from medical school rather than a devoted allegiance to the purpose of medical education, namely, the best care of each patient by a competent physician. A true physician focuses his or her care of each patient not only on the use of skilful and current techniques but also on the recognition of the unique needs and welfare of the patient. This professional devotion of the compassionate physician to the patient may be eroded as the concept of the oath faces challenges from the increasing demands and restrictions by corporate entities. The years of education and training lead to the agreement with a code of ethics in medicine that emphasizes behavior to earn the trust of patients. Some of the burnout of physicians may indicate the loss of autonomy and the need to free physicians to return to the core content of the oath, i.e., to uphold the highest standards of care for the safety and health of each patient.

## Review

Hippocrates wrote the Oath for Physicians about 2,500 years ago, and numerous translations and variations have emerged for medical students to take, usually at graduation time [1-2]. The actual date is between 600 before Christ (BC) and 100 anno Domini (AD), although frequently 450 BC is cited. In truth, there were many individuals involved in the creation of the Oath attributed to Hippocrates. That era of Western medicine had great changes so that an oath appeared necessary to protect the patients in those ancient times. The thrust of the Hippocratic Oath included not only the physician’s competence and dedication but also the critical needs of each patient [3]. The goal to protect the health of the patients continued, and the Declaration of Geneva was accepted by the World Medical Association (WMA) at the General Assembly of 1948 [4]. Numerous publications have called for amending the Oath since that time: many demands for changes increased after the Medical Device Amendments was signed into law in 1976 [5]. Most recently, on October 13, 2017, the WMA adopted a revised version of the substance to the Hippocratic Oath [6].

The people who took the Oath in ancient Greece did so to try to serve the best interests of the patients. This goal made medicine a profession rather than a trade. The tradesmen were “physicians” but with the goal of treating the rich and looking out for themselves. To counteract this problem, the Hippocratic Oath was given at the beginning of training rather than after the completion of studies and training—the latter timing in most of our medical schools of today. In fact, the concept of the Oath and serving the best interests of the patient define our standards today or, at least, what physicians still try to achieve for their patients. Modern challenges demand that physicians deal more and more with insurance companies and corporate medicine. As financial entities increasingly try to control physicians, the practice of medicine may become less and less of a profession able to achieve ideal goals for the individual patients. Medicine, as a noble profession, faces the conflicting forces of health economics on a daily basis and even with varying forms of “economic credentialing.”

At the same time as health care becomes more controlled and more infiltrated by businesses, the physician has been demoted to a "provider." And the definition of the doctor as a learned individual has become blurred with the other less extensively trained individuals in health care. More and more people talk of health care less as a noble profession and more as a business. At the same time as corporate influences in health care are increasing, there have been serious issues with transparency in the business world. Because of so many problems with the financial industries, Harvard Business School and some others have even developed a short oath. The latter encourages but does not require students to “take the Master of Business Administration (MBA) Oath” [7]. Many are deeply concerned that business school students and future leaders are losing the ability to empathize with people and need to focus on honesty and integrity. This lack of empathy contributed to the Madoff Scandal, the Financial Crisis, and other financial headlines with the victims of these schemes being ruined. Although the business school oath might suggest that those students are worthy of trust, it is not taken by many students. It remains unclear what the consequence would be of a violation. Interestingly, in 2015, an article questioned: “Is the MBA oath still relevant?” [8]. The article depicts the “model of economic man rational and self-interested.”  

In medicine, the Hippocratic Oath for physicians was written specifically to prevent self-interested doctors from harming individual patients in ancient times. To better serve the present day needs and current ethics of physicians, a more inclusive pledge was written and adopted in October 2017. One key addition is “I WILL RESPECT the autonomy and dignity of my patient" [6]. Also, “I will PRACTICE my profession with conscience and dignity” was increased to “and in accordance with good medical practice” [6]. These phrases offer an effort to address ethical principles that have been more fully included in two documents: 1) WMA’s Declaration of Helsinki: Ethical Principles for Medical Research Involving Human Subjects [9] and 2) the Declaration of Taipei on Ethical Considerations Regarding Health Databases and Biobanks [10]. Ultimately, the physician is dedicating his or her life and actions to the needs of the individual patient, as well as to humanity.

As the efforts continued to study the exact changes for the Oath of Physicians, a growing concern was the “burnout” of the medical profession. In this era, physicians' professional conduct emphasizes ethical standing and knowledge at the same time as society appears to value popularity in social media and poles about opinions. This difference in perspective of true quality based on study, training, and experience versus perceived value based on advertising and other business entities has resulted in confusion and stress for many physicians. There is even a worry that the Oath itself, i.e., that mindset of self-sacrifice, is contributing to the burnout. Currently, the updated version of the 2017 Revised Declaration of Geneva [11] contains the words: “I WILL ATTEND TO my own health, well-being, and abilities in order to provide care of the highest standard.” There was an interesting study to try to answer the question: "Does the Hippocratic Oath promote burnout?” This was published on March 28, 2017 by Medscape Business of Medicine [12]. The findings were that the 2,600 physicians studied were mixed in their feelings about the danger of burnout by putting patients first as required by their oath: 20% were unsure, 34% said it did, and 45% said it did not. Many felt the intrusion of corporate medicine, not their physician’s oath, was the culprit for burnout. 

There remains a huge divide in the philosophy of a business enterprise, rational to the point of little to no empathy and self-serving, and with a large investment in advertising strategies, versus that of physicians who deal with individual patient care and life and death matters. An additional problem lies in the different perspectives among doctors themselves when dealing with business organizations. This can lead to conflicts and decreased respect between colleagues of different viewpoints, i.e., some physicians agree more with the financial models than others. By the name change itself from an Oath to a Physician’s Pledge, perhaps there will be a difference in stress. At the same time, a pledge does not have the power in the word as does an oath. The latter word emphasizes that some principles in the care of the patient remain sacred.

## Conclusions

Ultimately, the world has many different cultures and values to words. Some graduating medical students have opted even to write their own oaths, a fact that means the pledge is to oneself and may or may not have the lasting impact as the original concept of Hippocrates. The question of the Hippocratic Oath and its many variations, of the Revised Declaration of Geneva Physician’s Pledge, and of personal oaths or pledges will continue to evolve as the perspectives on the value of the individual’s human life continues to change. Different cultures and pressures from changes in economic values will continue. One way to decrease burnout for future physicians may include discussion of the Hippocratic Oath at the beginning of medical school, as had been done in the era of Hippocrates. Then the medical students, who later swear in the graduation ceremony to his or her version of the Oath, will understand more fully the challenges that will be faced in a professional medical career. Medicine offers a life based on knowledge, skill, and service to the critical needs of each patient. No matter what the era or place of medical school, physicians must strive to maintain the goal of the profession, namely, to earn continued trust by protecting and treating safely the individual patient.
